# Extraocular Muscles Tension, Tonus, and Proprioception in Infantile Strabismus: Role of the Oculomotor System in the Pathogenesis of Infantile Strabismus—Review of the Literature

**DOI:** 10.1155/2016/5790981

**Published:** 2016-02-23

**Authors:** Costantino Schiavi

**Affiliations:** Department of Experimental, Diagnostic, and Specialty Medicine (DIMES), University of Bologna, St. Orsola-Malpighi Teaching Hospital, Via P. Palagi 9, 40138 Bologna, Italy

## Abstract

The role played by the extraocular muscles (EOMs) in the etiology of concomitant infantile strabismus is still debated and it has not yet definitively established if the sensory anomalies in concomitant strabismus are a consequence or a primary cause of the deviation. The commonest theory supposes that most strabismus results from abnormal innervation of the EOMs, but the cause of this dysfunction and its origin, whether central or peripheral, are still unknown. The interaction between sensory factors and innervational factors, that is, esotonus, accommodation, convergence, divergence, and vestibular reflexes in visually immature infants with family predisposition, is suspected to create conditions that prevent binocular alignment from stabilizing and strengthening. Some role in the onset of fixation instability and infantile strabismus could be played by the feedback control of eye movements and by dysfunction of eye muscle proprioception during the critical period of development of the visual sensory system. A possible role in the onset, maintenance, or worsening of the deviation of abnormalities of muscle force which have their clinical equivalent in eye muscle overaction and underaction has been investigated under either isometric or isotonic conditions, and in essence no significant anomalies of muscle force have been found in concomitant strabismus.

## 1. Ocular Motor Physiology

The extraocular muscles (EOMs) have structural characteristics and mechanisms of morphogenesis that are unique in mammalians. During the prenatal period, the development of eye muscles is mainly driven by genetic regulatory factors and it is highly dependent upon the development of brainstem ocular motor neurons [1, 2]. During the postnatal period the definitive muscle structure is established under the influence of epigenetic regulatory factors, that is, growth factors, hormones, and cell adhesion molecules [3, 4]. It has been hypothesized that there is a critical period for eye muscle development which lasts 3 to 6 months after birth, during which the eye muscles acquire the structure and function required by binocular vision but are more exposed to risks than in the adult [5]. During the critical period, the development of eye muscles is closely linked to the development of the visual sensory system. Muscle fiber composition in the EOMs in humans is more complex than in the other striated muscles, and EOMs are among the fastest and most fatigue-resistant muscles in the body. Rectus muscles consist of two separate layers: an inner global layer adjacent to the eyeball which extends the full muscle length and inserts at the sclera and an outer orbital layer adjacent to the periorbita which ends before the muscle becomes tendinous and inserts at the pulley. On the basis of morphologic data observed in light microscopy, that is, color and innervation pattern, and from the knowledge derived from physiological, biochemical, and molecular biology studies, six motor unit types may be recognized in the EOMs which differ from one another for fatigue resistance and fast twitch capabilities [5]. Both layers in the rectus muscles contain fast twitch and slow tonic fibers. The fast twitch muscle fibers have a single innervation site whereas slow muscle fibers have multiple innervation sites. It has not been possible to associate a specific type of eye muscle fiber with a specific type of eye movement. Anyhow, it is generally believed that slow eye movements and fixation mainly involve the slow eye muscle fibers [5]. Each motoneuron and its muscle fibers represent a final common pathway of the five eye movement systems, that is, vestibuloocular, optokinetic, pursuit, saccadic, and vergences, and all fiber types participate in every type of movement. The activity of single motor units is correlated with a given eye position, regardless of which type of eye movement subsystem is operating. Multiply innervated fibers in human horizontal eye muscles are not equally distributed between the medial and lateral rectus muscles. In fact, the number of slow fibers is the same in the global layers, whereas in the orbital layer it is 20% higher in the medial than in the lateral rectus muscle [6]. Nevertheless, isometric tension developed in response to succinylcholine, a depolarizing muscle relaxant that blocks singly innervated fibers of skeletal muscles but causes a long-lasting contracture of the multiply innervated eye muscle fibers, was found to be exactly the same in the two muscles, thus indicating that the amount of contractile components generating force in response to succinylcholine is the same in the two horizontal rectus muscles [7]. Succinylcholine activation of the multiply innervated fibers produces ocular alignment under general anesthesia that approximates that seen in the awake patient. These findings have suggested that the multiply innervated fibers may play a role in establishment of primary ocular alignment. Electromyography showed that the outer orbital layer fibers are recruited prior to those of the global layer, probably because they have to stabilize pulley position before global layer fibers begin to move the eyeball. It has been demonstrated with MRI that pulleys, that act for the functional origin of the EOMs, are not fixed structures but move posteriorly with eye muscle contraction and anteriorly with eye muscle relaxation [8, 9]. It is supposed therefore that they play an active role in ocular motility “active pulleys” but it is still debated if pulley stabilization during ocular rotations is only a passive response to eye muscle contraction or an active reaction driven by own musculature and innervation [9–12]. In paralytic strabismus, of either neurological or myogenic origin, the EOMs show typical alterations of either the gross anatomy or microscopic structure. In infantile concomitant strabismus instead, it is not generally believed that ocular misalignment is due to a primary anomaly of the eye muscles and/or the ocular motor system or any ocular motor subsystem. The gross anatomy of eye muscles and surrounding structures including the pulleys is apparently normal in concomitant strabismus. The histology of the eye muscle fibers was found also essentially normal, but changes have been observed in the muscle morphology, structure, and biochemical composition of the fibers, mostly in the singly innervated fibers of the orbital layer [13]. Functionally, this was found associated with slower contractions and reduced fatigue resistance of eye muscles in animals where monocular deprivation and strabismus were experimentally induced [14]. It is likely that these changes are secondary to the modified visual demands on the ocular motor control caused by the defects of binocular vision in strabismus from an early age. Adaptation of eye muscle function to visual demands could be also seen in the adult human ocular motor system, but here the effects could be reversed with treatment in some conditions.

The role played by the extraocular muscles in the etiology of concomitant infantile strabismus is still debated and it has not yet definitively established if the sensory anomalies in concomitant strabismus are a consequence of the misalignment or the primary cause of the deviation. It has been proposed that strabismus must be considered as possibly resulting from abnormal genetic and/or acquired factors and anatomical and/or functional abnormalities, in the sensory and/or the motor systems, both peripherally and in the brain itself [15]. The “weight” of each of these factors probably changes with the various types of strabismus.

## 2. Proprioception and Strabismus

The oculomotor system uses feedback control for short-term accuracy adjustments and as a long-term calibrator of ocular motor output. There are three sources of information from which the brain may determine where the eyes are pointing making it possible spatial orientation, egocentric localization, and calibration of eye movement commands: vision (outflow), efference copy (corollary discharge), and eye muscle proprioception (inflow). Vision provides accurate but long latency feedback control, matching the ocular movement with the visual target. A copy of the motor command (efference copy) provides higher neural centers with short latency information regarding the intended eye movement. Finally, eye muscle proprioception originating from muscle receptors provides information as to eye position changes. These muscle receptors are mainly represented by the palisade terminals or innervated myotendinous cylinders (IMCs) which are placed at the distal myotendinous junction [16–18].

Despite a number of studies on this topic, there is no general agreement on the specific function of IMCs in ocular motor control and visual spatial perception. Most authors believe that IMCs are sensory [18–21], but others have considered them as motor structures [22], or both [23]. In support to the “motor theory,” it has recently been demonstrated that these structures are cholinergic and that their origin lies in the oculomotor nuclei [22–27]. On the other hand, the location of IMCs at the myotendinous junctions and their continuity with tendon endings indicate a sensory function, putting the assumption of a pure motor function into question [24]. The primary afferent neurons carrying nonvisual inflow information from mammalian EOMs are located in the trigeminal ganglion [28–30]. The trigeminal innervation supplies muscle spindle afferents, at least in humans, whose eye muscles have muscle spindles and/or provides another as yet not known sensible innervation. Beyond the trigeminal ganglion, projections from palisade endings are then distributed in the central nervous system and thus it may be inferred that proprioception may be involved with visual and ocular motor processing at a number of levels [31–33]. The everyday adjustments of eye position from feedback control are typically short-term events that do not alter eye muscle structure. On the other hand, in the presence of extraocular muscle myopathies, paralytic or restrictive strabismus, the long-term adjustments needed to prevent diplopia and requiring constant increase in neuron discharge rate may cause muscle alterations, such as muscle hypertrophy and fiber type conversions. This is what happens in paralytic strabismus where the spread of comitance is the clinical corresponding sign. Although not generally accepted, proprioceptive feedback from eye muscle receptors is supposed to be involved in the control of ocular alignment and in the development of binocular vision, mostly during the critical period of development of the visual sensory system [34–37]. Although the role of eye muscle proprioception on the pathogenesis of concomitant infantile strabismus is not yet definitively known, several authors have attributed some forms of congenital concomitant strabismus to proprioceptive alterations [38–40]. Moreover, it has been proposed that alterations of eye muscle proprioception may be responsible for fixation instability in congenital nystagmus [41]. It has been demonstrated that the EOMs of strabismic patients have normal motor nerve endings, mainly located in the center of the muscle belly [42–44]. Conversely, ultrastructure of the distal myotendinous junction of the EOMs in congenital strabismus was found altered, thus indicating the presence of an altered proprioceptive innervation [40, 45]. Recently, these alterations of the distal myotendinous junction have been confirmed in patients with concomitant infantile strabismus but excluded in patients with adult onset concomitant strabismus [46]. Although the role of palisade endings is still not clear and their involvement in the inflow information is not definitively known, these morphologic data may suggest the hypothesis that an impairment of ocular proprioception in the myotendinous junction, most likely developed at an early age during the critical period of development of the visual sensory system, may play a role in the pathogenesis of infantile concomitant strabismus. In other words, a combination of failure of normal feedback signals from EOMs at an early age and immaturity of the visual sensory system may be suspected to be responsible for the development of infantile strabismus. The cause of a supposed primary defect of the myotendinous junction in the EOMs of patients with infantile strabismus is still unknown.

## 3. Extraocular Muscles Tonus

Innervational factors have been long since considered to be somehow implicated in the etiology and pathogenesis of concomitant strabismus, due to the close relationship between accommodation and convergence. Nevertheless, if refractive errors and anomalies of the accommodation mechanism may be suspected to be one of the causes of certain types of strabismus, it is evident that refractive errors and/or excessive or decreased accommodative convergence alone cannot explain the exact origin of concomitant strabismus. Other innervational factors have been considered trying to explain the etiology of concomitant strabismus. Among these, there is the progressive increase of esotonus, that is, the baseline innervation of the EOMs which opposes the normal position of rest in the awake state. Such rest position of the eyes consists of, for anatomical causes, a slight divergence and it is believed that in normal conditions binocular esotonus is superimposed upon the physiologic position of rest to keep the eyes roughly aligned. When for any cause binocular sensory input is altered early in life, monocular fixation could release an esotonus that leads the eyes to a convergent position and infantile esotropia takes shape [47]. The same happens when there is a unilateral visual loss from birth or with onset early in life, the so-called “functional monophthalmic syndrome.” In “functional monophthalmic syndrome” esotropia arises together with fixation preference in adduction, manifest latent nystagmus with a null zone in adduction, and a head turn toward the side of the viewing eye [48]. Essential infantile esotropia seems to be something like a double functional monophthalmic syndrome, since it can show fixation in adduction, manifest latent nystagmus, and head turn. According to Jampolsky [49, 50], esotonus should be distinguished from convergence since convergence is an active binocular function driven by binocular vision, whereas esotonus is a passive discharge that is released by the brain when fixation is monocular in the early phases of development. Esotonus has been proposed to be a “dissociated” phenomenon; that is, it can change in intensity leading to a change in horizontal ocular alignment that is unrelated to accommodation but is driven by changes in the balance of visual input from the two eyes [51]. “Dissociated esotonus” could also explain dissociated horizontal deviations and since “dissociated strabismus,” either horizontal or vertical, is typically associated with infantile convergent strabismus, one might infer that dissociated esotonus could be one of the causes of infantile esotropia [52]. If indeed esotonus due to monocular fixation early in life is the main cause of infantile esotropia, it remains still inexplicable why esotonus goes out of control in few patients only, those who become esotropic, while binocular alignment is unstable in most infants in the first weeks of life. Botulinum toxin, a natural neurotoxin that causes a flaccid paralysis of the muscle by inhibiting the exocytosis of the neurotransmitter-carrying vesicles and acetylcholine release in the neuromuscular junction [53], is widely used since the early eighties as a treatment of either concomitant or paralytic strabismus. The fact that single botulinum toxin injections may provide permanent correction of infantile esotropia, mostly when done at very early age [54–56], whereas the effect of the toxin on skeletal muscles is usually only temporary, due to functional reinnervation of the muscle after motoneuron sprouting, may have two explanations: one sensorial and the other purely motor. The temporary induced exotropia within a critical age breaks the esotonus and the sensory motor system resets itself, allowing orthotropia or small angle esotropia gradually to establish and to stabilize, given that a near orthotropia position is a more strong sensory motor condition anyway, compared to large angle esotropia [56]. The other explanation is supported by animal experiments which demonstrated permanent and more severe alterations after single botulinum injections in the orbital singly innervated fibers in infant and juvenile monkeys [57, 58]. These findings lead to two conclusions: first, the fiber type-specific alterations induced by botulinum toxin may permanently modify the function of the whole muscle, and second, since orbital singly innervated fibers are still maturing in the first 4–6 months after birth, this confirms the assumption that there is a critical period in the postnatal development of extraocular muscles.

## 4. Extraocular Muscles Tension 

**Figure 1:**
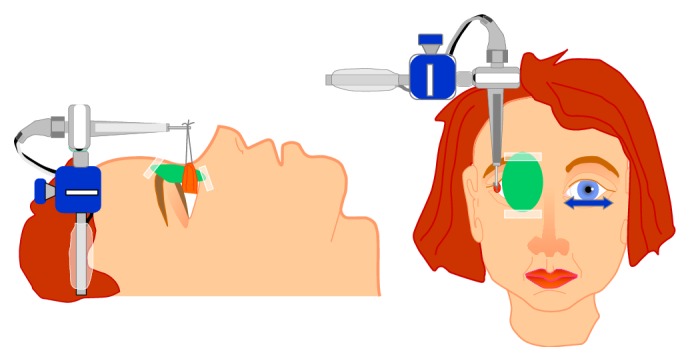
Tension measurement equipment: isometric muscle tension is recorded with a strain gauge system. A 5-0 silk suture is applied to the muscle tendon and tied around the strain gauge probe during strabismus surgery. Patient made saccadic movements with the nonrecorded eye (from [67]).

**Figure 2:**
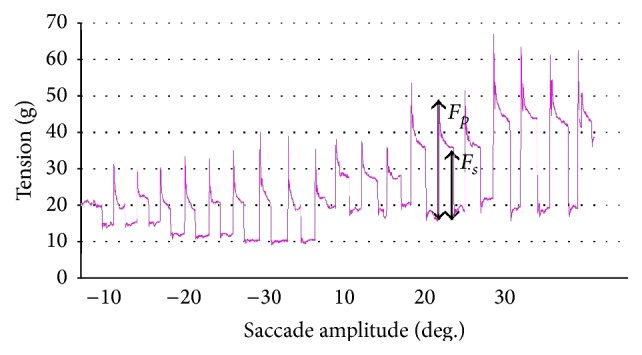
Tension recorded from a horizontal muscle of a strabismic patient who was making saccadic movements of 10° amplitude with the nonrecorded eye, from −30° in the off-direction to 30° in the on-direction of the recorded muscle. Deg. = degrees; g = grams; *F*_*p*_ = peak force; *F*_*s*_ = steady-state force (from [67]).

**Figure 3:**
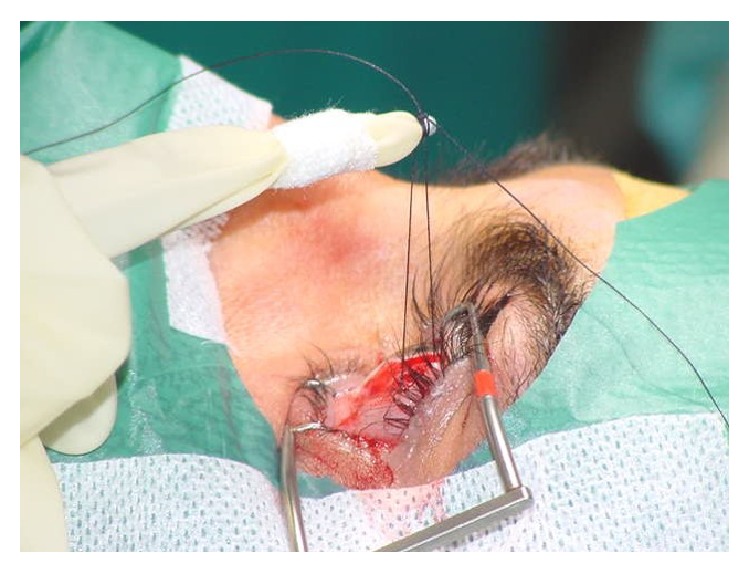
The probe is covered by a sterile surgical glove and tied up to the tendon of the lateral rectus muscle with a 5-0 silk suture during strabismus surgery under topical anesthesia.

As regards EOMs tension or force, it could be supposed that eye muscle force is involved in comitant strabismus, since from the clinical point of view EOMs may be overacting or underacting not only in paralytic strabismus but also in comitant strabismus. Tension developed by EOMs is coded by the motoneurons, as recently demonstrated in animal experiments [59], by the simultaneous recording of EOM tension with a muscle force transducer and motoneuronal firing rate in the alert cat preparation, so that any abnormality in muscle tension, for example, overforce, could be explained by some type of hyperactivation in motoneuronal firing, which in turn is driven by premotor brain neurons. The question is as follows: is muscle force altered in comitant strabismus? How is muscle force involved in comitant strabismus? If muscle force is altered in concomitant strabismus, an alteration of motoneuronal firing and therefore of premotor brain neurons could be suspected. Measurements of human extraocular muscle tension have been performed either indirectly with noninvasive techniques in either strabismic patients or healthy subjects or directly in strabismic patients undergoing strabismus surgery under topical anesthesia in both isometric and isotonic conditions by means of implanted force transducers or connecting the muscle tendon to a strain gauge (Figure 1). A sufficiently good concordance of the results obtained with direct and indirect techniques has emerged and such measurements have provided a considerable amount of information on the physiology and pathology of eye muscles. Levels of tension in the horizontal rectus muscles required to maintain fixation vary from a minimum of 8–12 g (grams) in the off-position of the muscle, that is, outside the field of action of the muscle, to a maximum of 75 g in the on-direction, that is, in the field of action of the muscle [60–63] (Figure 2). A peak of tension of the agonist muscle corresponds to the onset of a saccade, and then followed by a decrease of tension denoted steady-state tension at the end of the saccade (Figure 2). Active force of an eye muscle, that is, the force developed in extreme gaze, is significantly (26%) greater for the medial (75 g) than for the lateral rectus muscle (60 g) and a muscle which develops a maximum active force of less than 45 g would be suspected as paretic [63]. Isometric forces measured in horizontal eye muscles attached to or detached from the globe are the same. In other words, the muscle force development measured at the tendon is the same, no matter if the muscle is attached to or detached from the globe. Our group demonstrated this in 2003 [61, 62]. The equipment we used for eye muscle tension measurements consisted of a strain gauge probe connected through a silk suture with the muscle tendon still attached to or detached from the eyeball in patients undergoing strabismus surgery under topical anesthesia (Figure 3). Patients made saccades with the nonrecorded eye. Simultaneous tension recordings from the tendon and the pulley of horizontal eye muscles in patients undergoing strabismus surgery under topical anesthesia revealed that the force and the time course of force development are similar at the tendon and pulley of both the medial and lateral rectus muscles [64]. This would suggest that isometric force is simply passively transferred to the pulley by fibrous tissue surrounding the whole muscle belly rather than transmitted by the orbital fiber layer of the muscle directly inserted in the pulley. In fact, in this case one would expect lower force values at the pulley than at the tendon, as the ratio between number of muscle fibers in the orbital and global layers is around 2 : 3. This finding apparently conflicts with electromyographic results showing that the outer orbital layer fibers are recruited prior to those of the global layer and with the anatomic data of the insertion of the orbital layer of each rectus muscle on the pulley. Moreover, tension developed in response to succinylcholine, a depolarizing agent which paralyzes twitch muscle fibers of the body but activates slow fibers of EOMs, has the same amplitude in the medial rectus and lateral rectus muscles, despite a larger amount of slow fibers in the medial rectus orbital layer [6], as if the amount of fibers which respond to succinylcholine was similar in the medial rectus and lateral rectus muscles [7]. Succinylcholine activation of the multiply innervated fibers produces ocular alignment under general anesthesia that is similar to that seen in the awake patient, thus suggesting that the multiply innervated fibers of the orbital layer play a role in establishment of primary ocular alignment. We recorded simultaneously tension from the tendon and the pulley of the medial and lateral rectus muscles in response to succinylcholine activation in patients undergoing strabismus surgery under general anesthesia and we found that tension amplitude at the pulley is about 50% of amplitude at tendon, with no difference between the medial rectus and the lateral rectus muscles. These results do not permit to clearly establish if the pulley motor control is driven by the orbital layer alone or by fibrous tissue connections with the whole muscle belly [7–10]. Most of the studies done either with direct invasive measurements in strabismic patients [60, 63–65] or with indirect noninvasive measurements in strabismic patients [66] and in healthy subjects did not find significant differences of mechanical or contractile properties of horizontal eye muscles between patients with comitant strabismus and normal controls. Actually, there are few reports on differences of isometric tension of the horizontal muscles between strabismic patients and normals. In a study carried out in 2003 we compared tension values obtained in horizontal eye muscles during saccadic movements in patients undergoing strabismus surgery for comitant esotropia or exotropia under topical anesthesia and we found the same force development in the medial rectus and lateral rectus muscles, without differences between eso- and exotropic muscles [67]. In patients with exotropia, we found no differences between the medial rectus and lateral rectus muscles, whereas in esotropic patients the medial rectus seemed stronger than the lateral rectus. In that study we concluded that the force is the same in the medial and lateral rectus muscles and no differences exist between eso- and exotropic muscles in general, with the exception of the medial rectus in esotropic patients which seems to be somehow stronger than the lateral rectus [67]. Others found that the force developed by the medial rectus muscle was significantly greater in patients with esotropia, compared with normals and among patients with exotropia, those with intermittent exotropia had a force of the lateral rectus muscle close to that of the normal controls whereas it was significantly greater in those with constant exotropia than in the normals [68]. Moreover, the active force of the medial rectus muscle was found significantly smaller in patients with either intermittent or constant exotropia compared to normals [68]. Anyhow, these changes in muscle forces in patients with comitant strabismus seem to be the result of a long-lasting deviation rather than the cause or a joint cause of concomitant strabismus, as deep-seated ocular deviation may induce muscular hypertrophy leading to an increase of muscle force.

## 5. Conclusions

Eye muscle proprioception has been found in animal models to be implicated in modulating binocular functions during the critical period of development of the visual sensory system. In human infantile strabismus it has been demonstrated that the EOMs of strabismic patients have normal motor nerve endings but ultrastructural alterations on the distal myotendinous junction, the so-called IMCs, which have been considered long since the principal proprioceptors of human EOMs [40, 45]. These alterations have not been found in comitant strabismus of adult onset [46]. Although the exact role of IMCs is still not clear and recent evidences demonstrate that IMCs are motor rather than sensory structures, these ultrastructural anomalies of the myotendinous junction of the EOMs in congenital strabismus may indicate the presence of an altered proprioceptive innervation. These morphologic data may support the hypothesis that a disturbance of ocular proprioception in the myotendinous junction may play a role in the pathogenesis of concomitant infantile strabismus.

Esotonus not correctly driven by binocular vision during the early phases of development of the visual system has been proposed to be responsible for the establishment of convergent strabismus in early infancy, the so-called essential infantile esotropia syndrome [47, 52]. An impairment of binocular input to the two eyes should be therefore the primary cause. If so, it remains still unclear what this supposed impairment in binocular input is and why esotonus goes out of control in few cases only while most infants have uncoordinated eye movements in the first weeks of life. Moreover, if an uncontrolled esotonus is the cause or a joint cause of early infantile esotropia, one could suppose that esotonus can be defective in infantile exotropia where binocular functions are usually normal.

As far as eye muscle tension/force is considered, it seems to be essentially normal in comitant strabismus, with the exception of any imbalances between the MR and LR muscles and prevalence of one muscle or the other in patients with long-lasting comitant deviation, where muscle hypertrophy may take place [67, 68]. So muscle force/tension cannot be considered as a cause of comitant strabismus.

In conclusion, the role played by the EOMs in the etiopathogenesis of infantile concomitant strabismus is not yet fully clarified and the cause or causes of comitant infantile strabismus are not yet definitively known. It remains still inexplicable if the origin of infantile concomitant strabismus is primary sensory or motor.
